# Sensing when the wall comes tumbling down

**DOI:** 10.1093/jxb/eraa436

**Published:** 2020-10-16

**Authors:** David A Brummell

**Affiliations:** The New Zealand Institute for Plant and Food Research Limited, Private Bag, Palmerston North, New Zealand

**Keywords:** Comprehensive microarray polymer profiling, fruit softening, homogalacturonan, pectin, polygalacturonase, RNA-Seq

## Abstract

This article comments on:

**Paniagua C, Ric-Varas P, Garcia-Gago JA, López-Casado G, Blanco-Portales R, Muñoz-Blanco J, Schückel J, Knox JP, Matas AJ, Quesada MA, Posé S, Mercado JA**. 2020. Elucidating the role of polygalacturonase genes in strawberry fruit softening. Journal of Experimental Botany **71**, 7103–7117.

**Fruit ripening involves the activation of many genes, bringing about changes in colour, flavour, and texture. The plant cell can sense events occurring in its cell wall during pathogen invasion, but does it also sense what is happening to the wall as enzymes secreted into the apoplast cause its disassembly during ripening? Now,**  Paniagua *et al.* (2020)  **have combined three powerful tools to probe more deeply into the effects of polygalacturonase activity on cell wall disassembly and its feedback on gene expression.**

Excessive softening of fruit is a major cause of post-harvest losses throughout the world, contributing to wastage of up to half of the harvested crop (Porat *et al.*, 2018). Fruit losses during transport and storage and after-market in consumers’ homes would be substantially reduced if fruit softening could be controlled more precisely, while of course still allowing sufficient softening and textural change to provide a good eating experience. The commercial importance of this topic, let alone the environmental and societal implications, have necessitated much research activity over the past decades.

Fruit softening is a composite process that includes reductions in cell turgor and changes to the cell wall brought about by proteins secreted into the apoplast. The plant primary cell wall is composed of cellulose microfibrils embedded in a matrix made up of a variety of pectic and hemicellulosic polysaccharides and structural proteins, and in fruit is highly enriched in polyuronides [up to 60% of the wall (Redgwell *et al.*, 1997)]. On the outside of the walls of each cell is the middle lamella, composed largely of pectic homogalacturonan molecules, which serve as the glue that holds adjacent cells together. As fruit ripen, the cell wall and middle lamella are subjected to a programmed disassembly that cleaves polysaccharide backbones, removes side chains and methylester substitutions, alters the linkages between polysaccharides, and solubilizes some wall components (Brummell, 2006). One of the largest changes is a depolymerization and solubilization of the homogalacturonans of the middle lamella. The reduction in intercellular adhesion that this brings about helps result in fruit softening and textural change.

## Weakening the glue

Homogalacturonan molecules consist of long chains of galacturonic acid (GalA) residues, originally highly methylesterified and, together with rhamnogalacturonans, are part of large pectin macromolecules (Atmodjo *et al.*, 2013). Homogalacturonans can be depolymerized during ripening by either endo-polygalacturonase (PG; a reaction by hydrolysis) or by pectate lyase (PL; a reaction by β-elimination). Both PG and PL enzymes require a region of the homogalacturonan chain that has been de-methylesterified on a few GalA residues each side of the potential cut site, but the ultimate result, cleavage of the homogalacturonan chain, is the same. The action of the two enzymes can be distinguished since lysis by PL leaves a double bond at the non-reducing end of the cleaved polysaccharide.

The role of PG in fruit softening has been controversial since the pioneering antisense work of Smith *et al.* (1988) and Sheehy *et al.* (1988) in tomato, which unexpectedly found that silencing of the ripening-related and highly expressed *SlPG2* gene and reduction of its enzyme activity by 99% had only a minor effect on fruit softening (Kramer *et al.*, 1992). In contrast, a clear role for PG was evident in peach, where absence of the rapid melting component of softening was correlated with deletions or mutations of PG genes (Callahan *et al.*, 2004; Morgutti *et al.*, 2006). Subsequent antisense work also identified a role for PG-mediated homogalacturonan depolymerization in softening of strawberry and apple (Quesada *et al.*, 2009; Atkinson *et al.*, 2012). In the current work, Paniagua *et al.* (2020) have combined suppression of two strawberry PG genes, *FaPG1* and *FaPG2*, in the same transgenic line, and found that the effect of suppressing both genes on firmness was not additive relative to each alone. This implies that the two PG gene products may have slightly different functions in the wall. PL is the other enzyme capable of depolymerizing homogalacturonan during ripening, and in strawberry and tomato silencing of PL markedly diminished fruit softening (Jiménez-Bermúdez *et al.*, 2002; Uluisik *et al.*, 2016). There is evidence that in strawberry and tomato, PG and PL may target different pectic domains and/or be active in different parts of the wall (Posé *et al.*, 2015; Wang *et al.*, 2019), but strawberry and tomato may not be typical of all fruit. Peach, strawberry, and tomato all display ripening-related homogalacturonan depolymerization during ripening, yet the relative importance of the activities of PG and PL for softening appears to differ (Box 1).

Box 1. **The role of homogalacturonan depolymerization in fruit softening**

Fruit softening and textural change are determined partly by the original composition of the cell wall and partly by changes to the wall during ripening, both of which vary between species. In an unripe fruit (A, scale bar=200 nm), the middle lamella (ML) between the primary walls (PW) of adjacent cells is visible under the electron microscope. In ripe fruit (B), the middle lamella is degraded and essentially disappears. Depolymerization of homogalacturonan by PG or PL changes the size distribution of the molecules, from predominantly very large (C) to a greater preponderance of mid-sized and small molecules (D). This degradation weakens the ML, reducing intercellular adhesion and contributing to softening and textural change. Even if homogalacturonan depolymerization is diminished by gene editing of the major PG or PL, fruit will continue to soften to some extent (Wang *et al.*, 2019) due to the continuation of turgor changes and the action of remaining PGs and PLs and other ripening-related proteins, including expansins, β-galactosidases, α-arabinosidases, endo-1,4-β-glucanases, and xyloglucan endotransglycosylases/hydrolases (XTH).

In strawberry, suppression of either PG or PL reduced softening and resulted in pectins of higher molecular weight relative to the wild type (Posé *et al.*, 2015). It seems possible that different fruit species sit on a continuum for softening due to pectin degradation (E), between being mainly PG-mediated (melting-flesh peach), mainly PL-mediated (tomato), or somewhere in the middle (strawberry). If this is the case, where fruit from other species sit on the spectrum remains to be established.

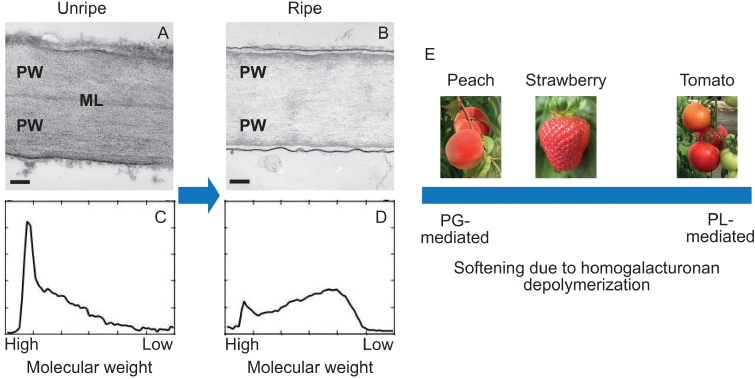

## How does cell wall modification feed back to affect the transcriptome?

Combining powerful techniques is likely to be the next wave in probing more deeply into biological processes. Paniagua *et al.* (2020) have investigated feedback between cell wall metabolism and the transcriptome by combining three such techniques. Suppression or overexpression by transgenic manipulation is the best way to determine the physiological role of a protein *in situ*. Transcriptome profiling by RNA-Seq provides a snapshot of the mRNA abundance of thousands of genes at a particular time, and comprehensive microarray polymer profiling (CoMPP) uses a set of monoclonal antibodies to assess the relative abundance of various polysaccharides in solubilized wall extracts. In the current work, cell walls were fractionated using a series of increasingly harsh solvents (water, sodium carbonate, KOH, and cadoxen), and CoMPP was used to determine the abundance in the extracts of particular polysaccharide epitopes, which are characteristic of different wall polymers. Changes to the solubility of a polysaccharide are indicative of the nature and the strength of its attachment to the bulk of the wall. Suppression of either *FaPG1* or *FaPG2* was sufficient to cause increased amounts of variously methylesterified homogalacturonans, rhamnogalacturonans, and arabinogalactan proteins in the later extracts, showing that these polymers were now more firmly attached to the wall. Surprisingly, highly methylesterified homogalacturonan became more weakly attached to the wall and appeared in the water extract.

Comparisons of gene expression in the transgenic lines found that strong suppression of one PG gene did not necessarily mean strong suppression of the other, even though the two genes both encode PGs (see Box 2). Suppression of both PG genes had a remarkable effect on the transcriptome, down-regulating a range of other genes encoding cell wall-modifying proteins, including pectin methylesterases, β-galactosidases, endo-1,4-β-glucanases, an expansin, and genes involved in cell wall biosynthesis and protein processing. Similarly, silencing of tomato *SlPL* reduced the expression of genes encoding expansin, PG, and xyloglucan endotransglycosylase/hydrolase (XTH) (Yang *et al.*, 2017), and overexpression of the persimmon ripening-related gene *DkXTH8* in transgenic tomato altered fruit wall structure, increased the mRNA abundance of the ethylene biosynthesis genes *SlACS2*, *SlACS4*, and *SlACO1*, advanced the ethylene climacteric, and hastened ripening and softening (Han *et al.*, 2016). The challenge now is to understand how cell wall restructuring feeds back to cause changes in the transcriptome, and the consequent changes that result from these alterations in gene expression.

Box 2. **A feedback mechanism from the cell wall for altering gene expression**

Paniagua *et al.* (2020) found that suppression of two ripening-related PG genes resulted in the altered expression of a range of genes, including those encoding other cell wall-modifying proteins of completely different classes. The implications of this are that the cell is able to sense what is happening in the wall, and adjust the expression of other genes accordingly. This provides evidence to help explain a perplexing older example of this phenomenon, as shown here (re-compiled from Brummell and Harpster, 2001). In these tomato plants, mRNA of the ripening-related expansin gene *SlEXPA1* was suppressed by constitutive expression of a sense transgene (Brummell *et al.*, 1999), and was virtually undetectable in the suppressed lines (A, B). The mRNA abundance of three other expansin genes (with some DNA sequence similarity to *SlEXPA1*), expressed mainly prior to ripening, was not affected (A). In contrast, mRNA abundance of the major ripening-related PG gene *SlPG2* (with no DNA sequence similarity to *SlEXPA1*) was reduced in the *SlEXPA1*-suppressed fruit by >75% relative to the control (B). How does the cell know that wall modification has been made? Is sensing related to the transcription or mRNA turnover of particular cell wall genes, amounts of proteins or polysaccharides transiting to the wall via the Golgi apparatus, detection of solubilized apoplastic cell wall (pectic?) fragments by plasma membrane-localized receptors, or some other mechanism?

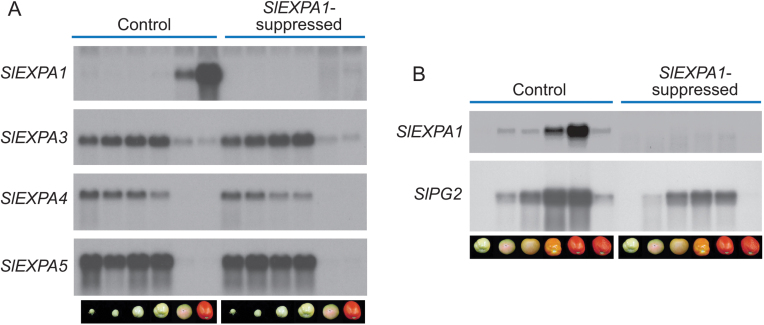

## Are cell wall modification and loss of turgor linked?

A reduction in cell turgor accompanies ripening and is a component of fruit softening and textural change (Shackel *et al.*, 1991). The loss of turgor results from two processes, the movement of solutes to the cell wall space and consequent water movements, plus post-harvest whole-fruit water loss (Saladié *et al.*, 2007; Wada *et al.*, 2008). Paniagua *et al.* (2020) identify a transcript for a putative high-affinity potassium transporter that was up-regulated to high abundance when PG genes were suppressed, and speculate that this gene product may be involved in the solute movements that result in turgor change. Further work is needed, but this would imply that the cell is able to sense events occurring in the cell wall and modify gene expression to control a different component of the ripening process. Interestingly, a connection between cell wall modification and turgor was also made in apple, where silencing of the ripening-related *MdPG1* gene unexpectedly but dramatically reduced post-harvest whole-fruit water loss (Atkinson *et al.*, 2012). This study also provided evidence that transgenic manipulation of the expression of a single PG gene resulted in changes to physiological parameters related to water status. The complex relationships between the wall modification and turgor loss aspects of softening and textural change are an exciting area for further study.
